# Optimizing a feasible protocol for acellular nerve allografts: An experimental study

**DOI:** 10.1007/s10561-025-10189-w

**Published:** 2025-10-30

**Authors:** Marta de Juan Marín, Marta Pevida, Sara Llames, Juan Argüelles Luís, Daniel Camporro Fernández, Álvaro Meana

**Affiliations:** 1 Department of Plastic and Reconstructive Surgery, Central University Hospital of Asturias, Oviedo, Spain; 2 Department of Functional Biology, Faculty of Medicine and Health Sciences, University of Oviedo, Oviedo, Spain; 3 Unidad de Ingeniería Tisular, Centro Comunitario Sangre y Tejidos de Asturias (CCST), 33006 Oviedo, Spain; 4 Instituto de Investigación Sanitaria del Principado de Asturias (ISPA), 33011 Oviedo, Spain; 5 Centro de Investigación Biomédica en Red en Enfermedades Raras (CIBERER) ISCIII, 28029 Madrid, Spain; 6 Instituto Universitario Fernández-Vega, Fundación de Investigación Oftalmológica, Universidad de Oviedo, 33012 Oviedo, Spain; 7 Instituto de Investigación Sanitaria-Fundación Jiménez Díaz (IIS-FJD), 28015 Madrid, Spain; 8 Department of Functional Biology, Neuroscience Institute of Asturias (INEUROPA), Faculty of Medicine and Health Sciences, University of Oviedo, Oviedo, Spain; 9 Present Address: Clínica Alejandría, C/Sorní 12 Bajo. C.P., 46004 Valencia, Spain

**Keywords:** Acellular nerve allografts, Decellularized nerve allografts, Peripheral nerve regeneration, Nerve tissue engineering, Muscle histomorphometry

## Abstract

Peripheral nerve injuries often require surgical intervention when end-to-end coaptation is not feasible, with autologous nerve grafts being the current gold standard. However, limitations such as donor-site defects drive the search for alternative methods. This study explores the efficacy of acellular nerve allografts obtained through a feasible protocol as a potential off-the-shelf substitute for autografting in a 14-mm rat sciatic nerve defect. Thirty-two female Wistar rats were divided into four groups: autograft, lyophilized acellular allograft, fresh acellular allograft and silicone tube. Functional assessments and histological examinations were performed at 14 and 20 weeks post-surgery, respectively. Results showed comparable axonal regeneration between acellular nerve allografts and autografts. Histomorphometric analysis revealed no significant differences in axonal characteristics between groups. Muscle histomorphometry indicated superior recovery in animals treated with fresh acellular allografts, who exhibited the least muscle atrophy and larger muscle fiber diameter compared to lyophilized processed allografts and autografts. Functional assessments revealed no significant intergroup differences. Processed acellular allografts promote axonal regeneration similar to autografts in a 14-mm rat sciatic nerve defect. Fresh acellular allografts achieve better muscle reinnervation in the medial gastrocnemius muscle. However, axonal regeneration does not consistently correlate with functional or histomorphological outcomes of the hind leg muscle. The successful decellularization protocol and lack of immune rejection pave the way for adapting it to human nerve grafts. These could revolutionize clinical practice in our country, becoming an example of leveraging existing resources and replacing collagen conduits and autografts for treating certain injuries.

## Introduction

The term "peripheral nerve" refers to nerves located outside the brain and spinal cord, whose function is to connect the central nervous system with the limbs and organs of the body. Their injuries can range from brachial plexus paralysis to isolated lesions of a single nerve, with causes including trauma, iatrogenesis, tumors, compression or causes of unknown origin. The appropriate treatment depends on the time elapsed since the injury, the type, and the location thereof.

Peripheral nerve injuries affect approximately 3% of trauma patients, and in the United States alone, over 200,000 nerve injuries occur annually as a result of trauma (Isaacs 2013; Ives et al. 2017; Noble et al. 1998; Robinson 2000; Szynkaruk et al. 2013). Additionally, worldwide, over 1 million amputations are performed each year, with a projected increase to 3.6 million by 2050, often leading to chronic pain in the residual limb or phantom limb pain (Dumanian et al. 2019; Ives et al. 2017).

Despite research in neuroscience and surgery, improving functional outcomes after repairing nerve injuries remains a challenge. In complex cases, the standard treatment involves the use of autologous nerve grafts or nerve transfers when direct tension-free suturing cannot be performed. However, these methods have limitations and disadvantages, leading to the search for nerve substitutes (Pan et al. 2020).

Substitutes that allow nerve regeneration have been developed, such as processed acellular nerve allografts and guide conduits. These allografts maintain an organized extracellular matrix that promotes cell migration and angiogenesis and have shown to be superior to guide conduits in terms of nerve regeneration (Szynkaruk et al. 2013; Wood et al. 2011).

The process of obtaining acellular nerve allografts involves decellularization techniques, such as the use of detergents and enzymes, and has become a popular alternative to autologous nerve grafts in certain cases (Johnson et al. 1982; Moore et al. 2011; Sondell et al. 1998). However, the challenge remains to strike a balance between treatment efficacy and associated costs.

Nerve repair ultimately depends on various variables, including the distance axons must travel, the axonal regeneration capacity over time, the size of the defect and the nature of the nerve. Although autologous nerve grafts typically yield the best results, there are clinical situations where alternatives such as acellular allografts are preferred due to considerations such as the additional damage caused by autograft extraction (Gordon et al. 2009; Kubiak et al. 2018; Wood et al. 2011; Wood and Mackinnon 2015).

Current clinical indications for the use of acellular nerve allografts include grafting non-critical sensory nerve defects, treating chronic limb pain or phantom limb pain, extension grafts in sensory transfers, and distal defects in mixed nerves (Hong et al. 2019; Sachanandani et al. 2014; Safa and Buncke 2016; Saheb-Al-Zamani et al. 2013; wamsleyk 2016).

In summary, acellular nerve allografts represent a promising option in the repair of peripheral nerve injuries, offering advantages over autografts in certain cases.

This study aims to explore alternatives to autologous nerve grafts for repairing segmental defects in peripheral nerves, while considering the constraints and opportunities within the public health system and available resources. Regeneration achieved in a peripheral nerve defect repaired with acellular nerve allografts was compared against the current standard technique, autologous nerve grafts. The main objective is to compare nerve regeneration between both methods using histomorphological variables of the nerve, muscle histomorphology, and functional tests. Specific objectives include laying the groundwork for developing human acellular nerve allografts and comparing different preservation methods of nerve allografts to make them more accessible and easier to commercialize. Additionally, developing a semi-automated histomorphological analysis tool for peripheral nervous system studies is included.

## Materials and methods

### Experimental design

We conducted an experimental study involving 32 adult female Wistar rats weighing between 190 and 280 g (Wistar IGS Rat, Charles River). These rats were randomly assigned to four experimental groups, each consisting of 8 rats. The study aimed to investigate the efficacy of four different surgical techniques in repairing a 14 mm unilateral defect in the sciatic nerve.

- Group A: Positive control, where a nerve autograft was utilized for nerve repair.
- Group B: Utilized for repair.
- Group C: Employed freshly preserved decellularized nerve allograft.
- Group D: Negative control, where a silicone tube was used for repair.

At 14 weeks post-surgery, functional assessments were conducted on each group, followed by euthanasia at week 20 for histological and morphological examination of the repaired sciatic nerve and associated muscles compared to the healthy limb. All procedures involving animals adhered strictly to EU Directive 2010/63/EU and received approval from the Ethical Committee for Animal Experimentation of the University of Oviedo, Spain.

### Surgical procedures

Anesthesia induction was achieved via intraperitoneal injection of ketamine hydrochloride 75 mg/kg (Imalgene®; Merial Laboratorios S.A., Barcelona) and xylazine 5 mg/kg (Rompun®, Bayer Hispania S.L., Barcelona), followed by subcutaneous administration of carprofen 5–10 mg/kg (Rimadyl®, Zoetis Spain S.L., Madrid) and buprenorphine 0.2–0.5 mg/kg (Bupaq®, Laboratorios Karizoo S.A., Barcelona) for pain management. Anesthesia was maintained with continuous inhalation of isoflurane 1.5% (Isoflo®, Esteve Veterinaria, Ciudad Real). Antibiotic prophylaxis was provided using enrofloxacin 2.5% (Alsir®, Ecuphar Veterinaria S.L.U., Barcelona) at a dose of 5 mg/kg body weight every 12 h for 1 day. Surgical procedures were performed under a surgical microscope by a single investigator.

Recipient animals were positioned prone, and their lower extremities were abducted. A skin incision was made to expose the sciatic nerve, followed by transection and creation of a 14 mm nerve gap. Each animal was treated according to the group assigned as stated above. Post-suture, the area was irrigated with saline, and muscle fascia and skin were closed. Animals were allowed to recover before returning to their cages.

### Preparation of detergent-processed allograft

In the process of preparing allografts for groups B and C, the sciatic nerve was harvested from both hind legs of Wistar rats weighing between 200 and 350 g. These animals are commonly utilized in experimental microsurgery procedures at the Plastic Surgery Department of the Central University Hospital of Asturias.

The procedure was carried out following the technique described previously (“surgical procedures”). After shaving the hind limbs and ensuring sterile conditions, an incision was made from the coccyx to the lateral femoral condyle to expose the femoral biceps. Gentle dissection separated the femoral biceps and the superficial gluteus to expose the sciatic nerve from its exit point to its trifurcation at the knee level.

Grafts were obtained from the area immediately distal to the femoral biceps' exit point up to 5 mm proximal to the nerve trifurcation at knee level. This procedure was performed bilaterally. Subsequently, the grafts were promptly immersed in a sterile container filled with physiological saline solution (NaCl 0.9%, B. Braun, Barcelona) and stored at 4–8 °C. Finally, donor animals were euthanized via intracardiac injection of sodium pentobarbital 100 mg/kg (Dolethal, Vétoquinol E.V.S.A., Madrid).

*Decellularization protocol.* Within less than 24 hours (h), the grafts underwent an incubation process with a 1M hypertonic NaCl solution (Sigma Aldrich Inc.) moderate agitation and room temperature for 24 h. Subsequently, the sample was washed with 0.1% sodium dodecyl sulfate (SDS) (Sigma Aldrich Inc.) and 1× phosphate-buffered saline (PBS) (Thermo Fisher Scientific) under constant moderate agitation and room temperature for 3 days. The obtained grafts were allocated to experimental groups B and C, with preservation methods varying between the two. In the case of freshly preserved decellularized allografts, they were stored in a sterile container with saline solution (NaCl 0.9%, B. Braun, Barcelona) at 4 °C after processing. For lyophilized decellularized allografts, they were frozen at − 80 °C for 24 h and then subjected to lyophilization (LyoQuest, Telstar®). Subsequently, they were kept in a sterile container without any solution at room temperature storage.

### Functional assessment

At 14 weeks post-surgery, motor function was assessed using the sciatic functional index, tibial functional index, peroneal functional index (Bain 1998; de Medinaceli et al. 1982), and Rotarod test. The Rotarod test evaluated motor coordination and endurance (Hamm et al. 1994). Animals underwent training a day prior to the final test, which involved accelerating from 4 to 40 rpm over 10 min. Latency and speed were recorded, with four trials conducted per rat on the same day.

### Nerve histomorphometry

Histological analysis of nerves was performed at week 20 post-surgery. Nerves were fixed, post-fixed, sectioned, and stained for light microscopy imaging and quantitative analysis. Fiji software was utilized for nerve fiber count (Wood et al. 2011), density, axon count, fiber diameter, neural area, myelin thickness and percent neural tissue (Martins et al. 2006; Schindelin et al. 2012; Schneider et al. 2012).

### Muscle histomorphometry

Histological analysis and wet muscle mass measurement were performed on reinnervated muscles at week 20. Muscles collected were the medial gastrocnemius muscle and the tibialis anterior muscle. Muscle mass ratio between experimental and healthy sides was calculated to assess muscle atrophy. Muscle samples were stained and classified based on fiber diameter, presence of central nuclei, collagen infiltration, and degree of fatty degeneration. Muscle impact was categorized as mild, moderate or severe based on a scoring system.

### Statistical analysis

Data were analyzed using IBM SPSS Statistics for Windows, version 22 (IBM Corp., Armonk, N.Y., USA). As the sample size was small, non-parametric tests were employed. Quantitative variables were reported as median and interquartile range, while qualitative data were presented via frequency distribution tables. Kruskal–Wallis test was used for each variable and nerve segment. Box plots were prepared for variables with *p* value < 0.1, and Holm-Bonferroni adjustment was applied for multiple contrasts. Mann–Whitney test was conducted for significant variables, with subsequent Holm-Bonferroni adjustment for group pairings. The use of non-parametric tests, such as the Mann–Whitney test, in this study is justified by the small sample size, the potential lack of normality in the data, the ordinal or non-continuous nature of the variables, and the preference for reporting medians, which provides a more appropriate and reliable statistical comparison under these conditions.

## Results

### Animals

During the initial surgery and within the first 24 h post-surgery, five experimental animals were lost. Three died within the early postoperative period due to unknown causes, one sustained severe self-inflicted injuries to the treated hind limb and the last experienced irreparable wound dehiscence on the day following surgery. End-point criteria were applied in all cases. To maintain the sample size of 8 animals in the control group, an additional Wistar rat was included, resulting in a final sample size of 33 experimental animals, with a 15% loss rate (n = 5).

### Functional assessment

**Fig. 1:**
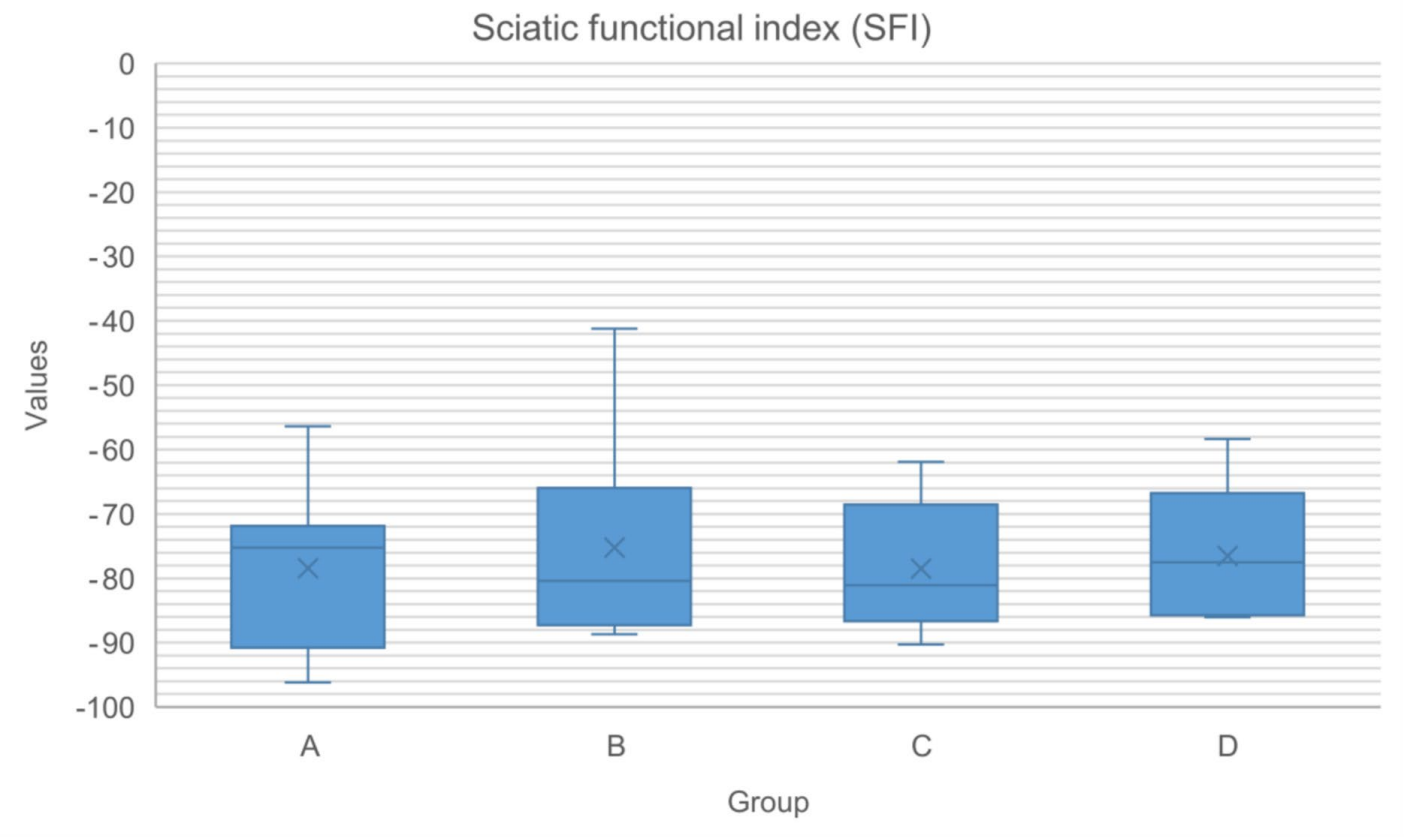
Box-plot representing the values of the sciatic functional index within each group

**Fig. 2:**
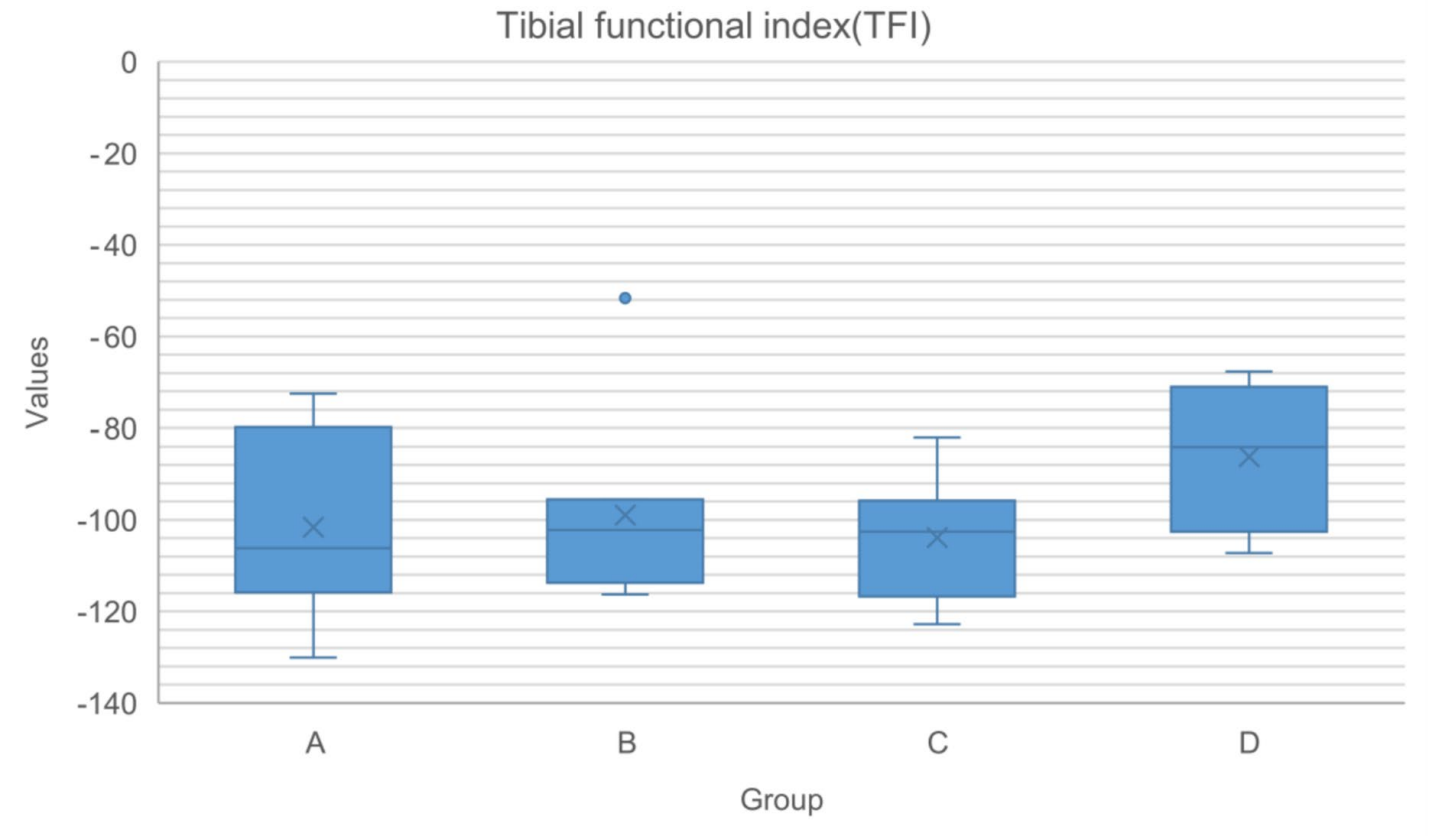
Box-plot representing the values of the tibial functional index within each group

**Fig. 3:**
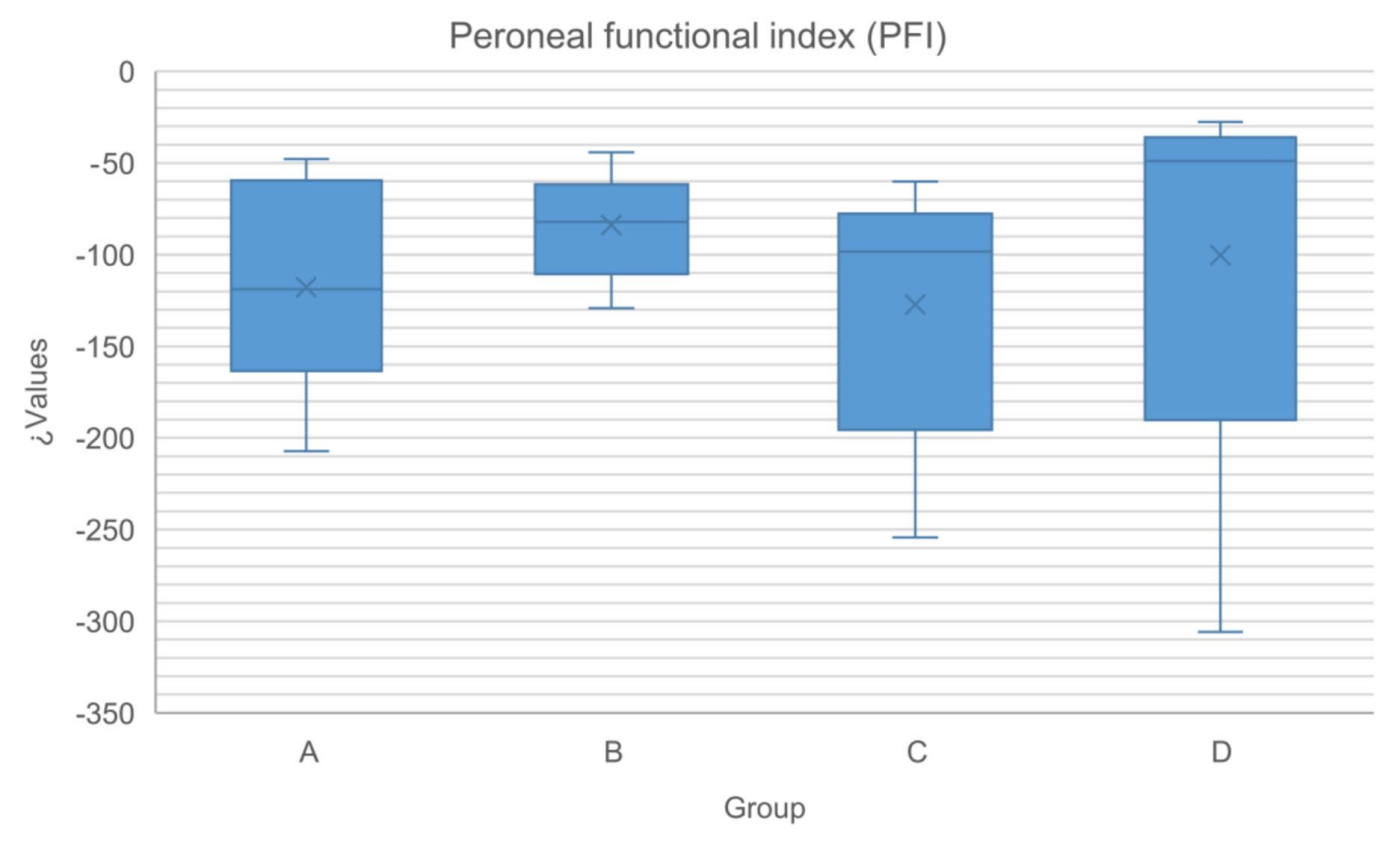
Box-plot representing the values of the peroneal functional index within each group

Footprint analysis of hind limbs revealed that the distance between the second and fourth toes could not be measured and was assigned a predetermined value of 6 mm for all experimental footprints (right hind limb). Analysis of sciatic, peroneal, and tibial functional indexes showed no significant differences between groups, with the highest values observed in the sciatic functional index for groups A, B, and C, and the peroneal functional index for group D (Table 1, Figs. 1, 2 and 3).

**Table 1:** Sciatic, tibial and peroneal functional indexes at week 14 postoperatively for each experimental group (A–D)

Group (n)	SFI		TFI		PFI	
	Median	Range	Median	Range	Median	Range
A (8)	− 75.25	− 96.20 to − 56.44	− 106.21	− 130.10 to − 72.48	− 118.86	− 207.19 to − 47.87
B (7)	− 80.42	− 88.70 to − 41.21	− 102.18	− 116.27 to − 51.60	− 82.14	− 129.06 to − 44.07
C (8)	− 81.09	− 90.30 to − 61.86	− 102.54	− 122.83 to − 81.96	− 98.30	− 254.33 to − 60.02
D (5)	− 77.49	− 86.06 to − 58.32	− 84.07	− 107.27 to − 67.65	− 48.91	− 305.76 to − 27.55

In brackets are sample sizes available for functional assessment. SFI, sciatic functional index. TFI, tibial functional index. PFI, peroneal functional index

**Fig. 4:**
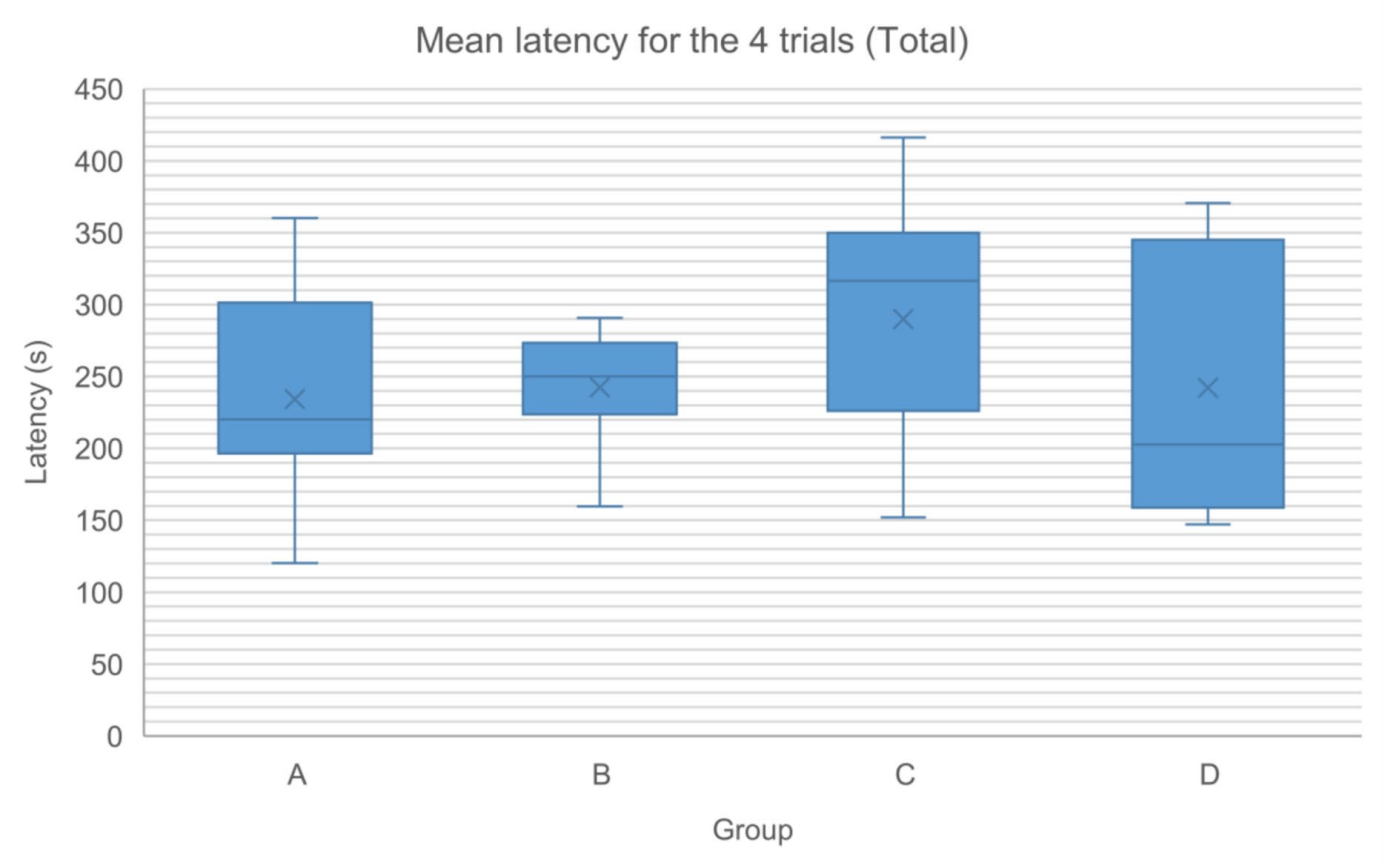
The mean latency value (s) of the four trials for each experimental group (Total)

**Fig. 5:**
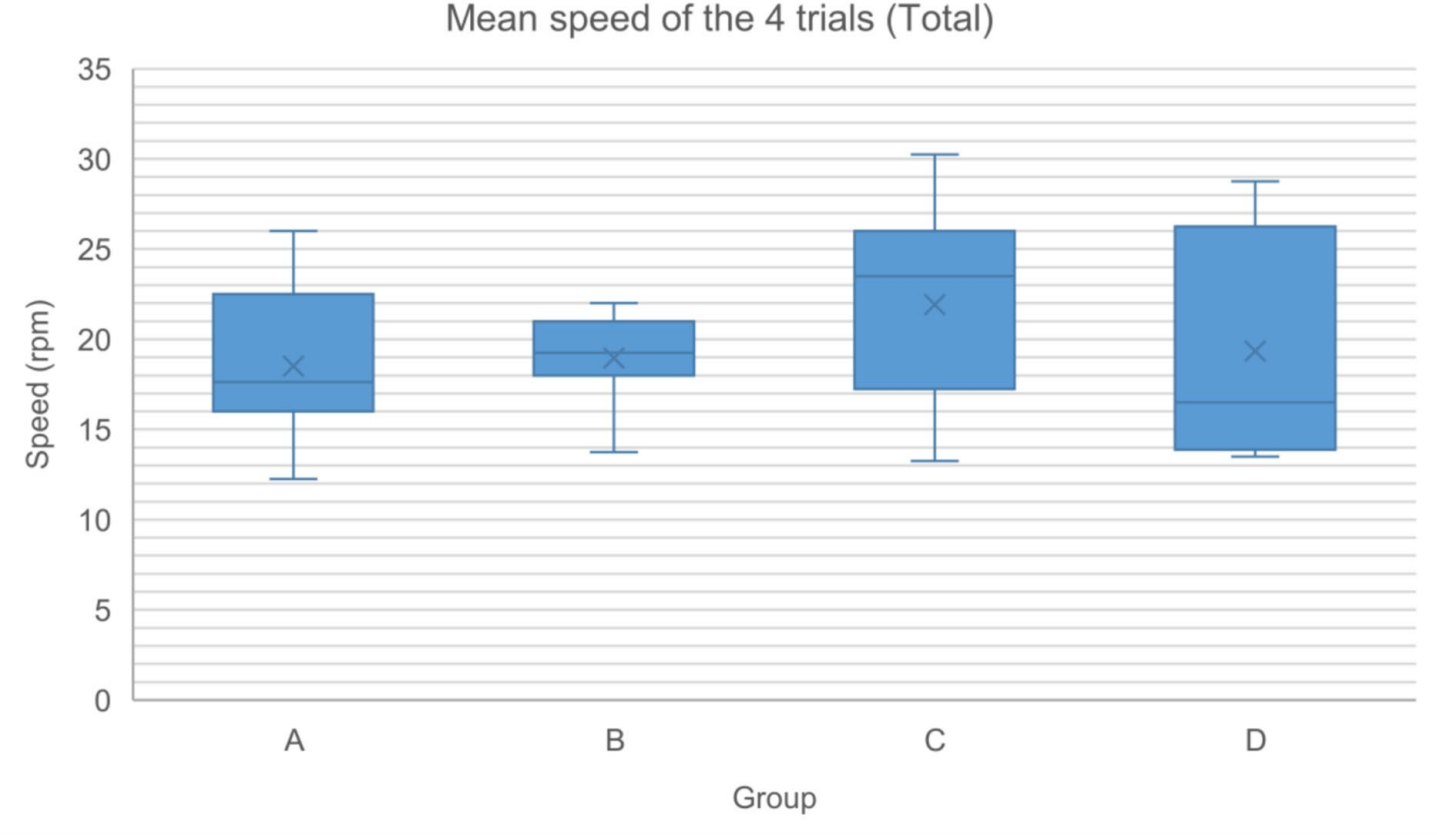
The mean speed value (rpm) of the four trials for each experimental group (Total)

The Rotarod test demonstrated a positive correlation between trials and latency time/complementary speed. However, no significant differences were found between groups in terms of latency time and complementary speed across trials (Tables 2, 3, Figs. 4 and 5).

**Table 2:** Latency (s) at the Rotarod test at each trial (T1–T4) and the mean latency value of the four trials for each experimental group (Total)

Group (n)	T1 (s)		T2 (s)		T3 (s)		T4 (s)		Total (s)	
	Median	Range	Median	Range	Median	Range	Median	Range	Median	Range
A (8)	192.50	62.00–324.00	234.00	152.00–297.00	209.00	148.00–399.00	268.50	119.00–497.00	220.13	120.25–360.25
B (7)	209.00	194.00–263.00	238.00	163.00–301.00	300.00	149.00–323.00	294.00	126.00–306.00	250.00	159.50–290.75
C (7)	208.00	140.00–382.00	355.00	100.00–468.00	336.00	147.00–386.00	300.00	200.00–521.00	316.50	152.00–416.25
D (5)	221.00	157.00–322.00	203.00	104.00–355.00	162.00	150.00–470.00	237.00	169.00–421.00	202.75	147.25–370.50

**Table 3:** Speed (rpm) at the Rotarod test at each trial (T1–T4) and the mean speed value of the four trials for each experimental group (Total)

Group (n)	T1 (rpm)		T2 (rpm)		T3 (rpm)		T4 (rpm)		Total (rpm)	
	Median	Range	Median	Range	Median	Range	Median	Range	Median	Range
A (8)	16.00	12.00–24.00	18.50	13.00–22.00	17.00	13.00–29.00	20.50	11.00–35.00	17.63	12.25–26.00
B (7)	17.00	16.00–20.00	18.00	14.00–23.00	23.00	13.00–24.00	22.00	12.00–23.00	19.25	13.75–22.00
C (7)	17.00	12.00–28.00	27.00	10.00–33.00	25.00	13.00–27.00	21.00	16.00–37.00	23.50	13.25–30.25
D (5)	18.00	14.00–24.00	16.00	10.00–26.00	14.00	13.00–33.00	19.00	14.00–37.00	16.50	13.50–28.75

### Nerve histomorphometry (Table 4)

**Table 4:** Histomorphometrical variables of a healthy sciatic nerve of the left hindlimb of 9 Wistar rats to use as a reference

Variables	Median	Minimum	Maximum	Mean	Standard deviation
Fiber Density (n/100 µm^2^)	1.48	0.80	1.82	1.39	0.29
Axons (n)	3710.50	1992.00	4561.00	3481.83	737.03
Fiber width (µm)	3.81	3.07	4.51	3.84	0.54
Axonal area (µm^2^)	18.41	15.34	35.29	22.21	7.03
Myelin thickness (µm)	15.94	12.43	20.81	15.93	3.16
Percentage nerve tissue (%)	27.99	22.44	37.68	29.39	5.31

**Fig. 6:**
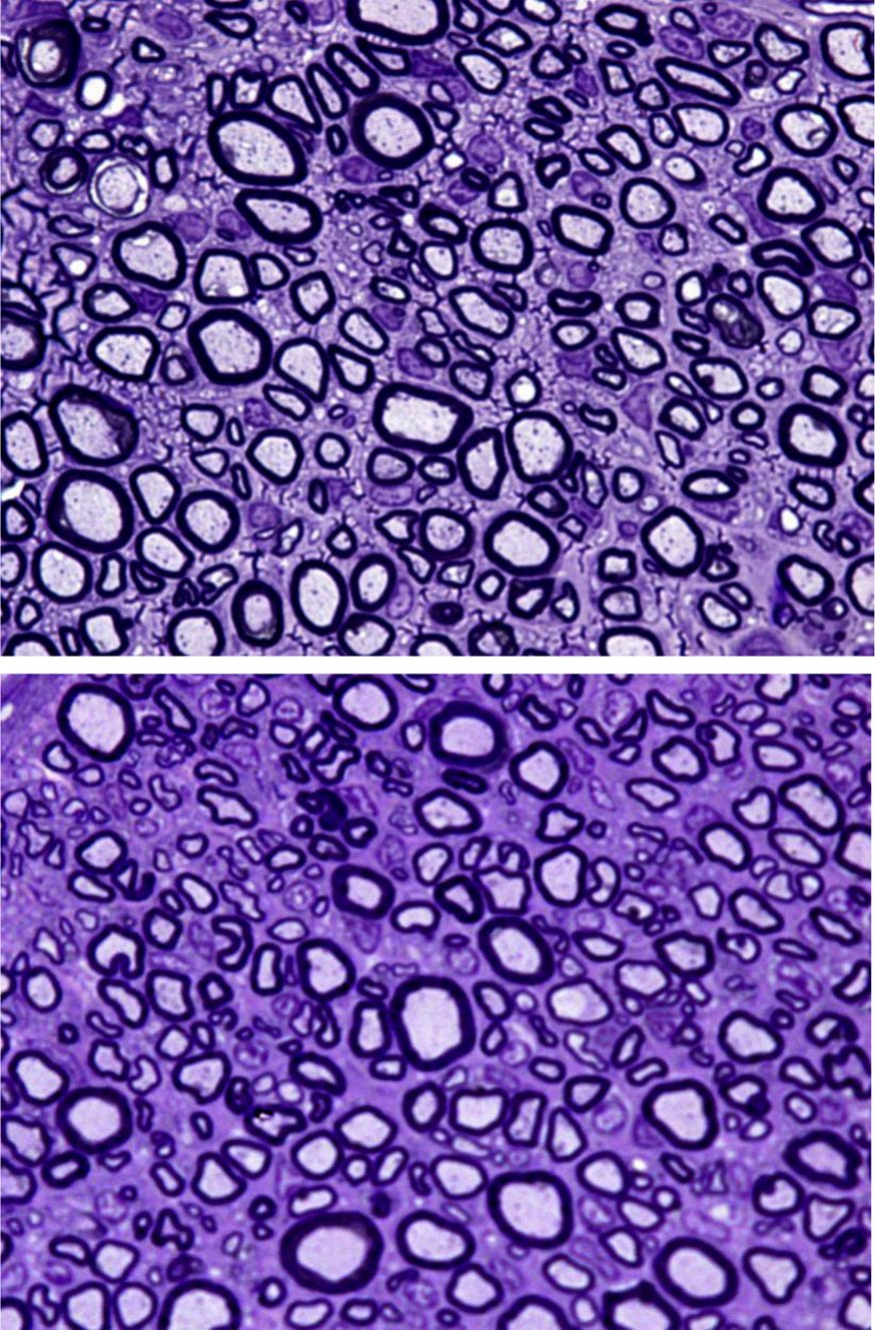
In the image above, a semithin cross-section of the **distal segment** of the sciatic nerve from a rat in **group A**, stained with toluidine blue and viewed at ×1000 magnification. In the imagen below, a semithin cross-section of the **middle segment** of the sciatic nerve from the same animal, stained with toluidine blue and viewed at ×1000 magnification

**Fig. 7:**
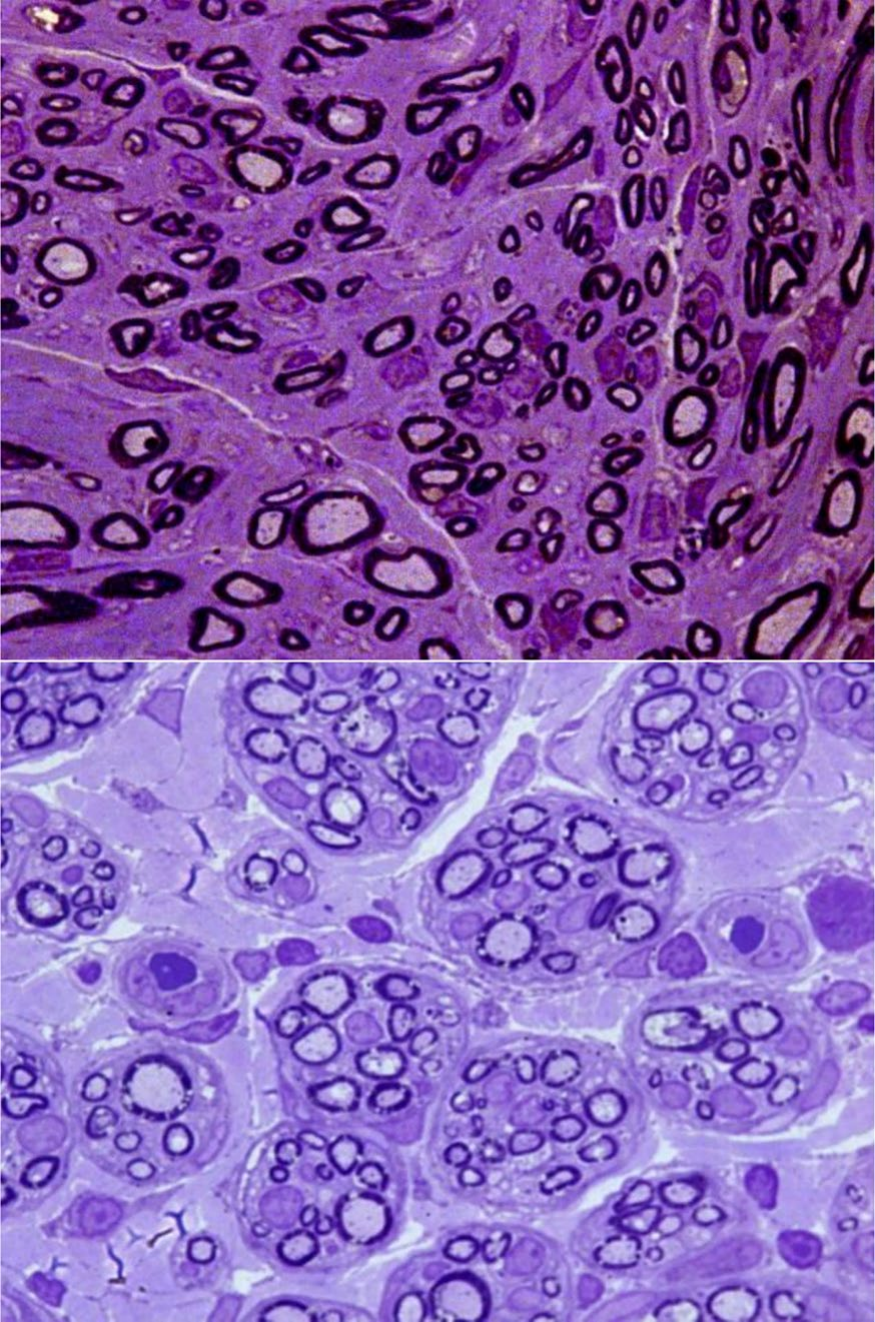
In the image above, a semithin cross-section of the **distal segment** of the sciatic nerve from a rat in **group B**, stained with toluidine blue and viewed at ×1000 magnification. In the image below, a semithin cross-section of the **middle segment** of the sciatic nerve from the same animal, stained with toluidine blue and viewed at ×1000 magnification

**Fig. 8:**
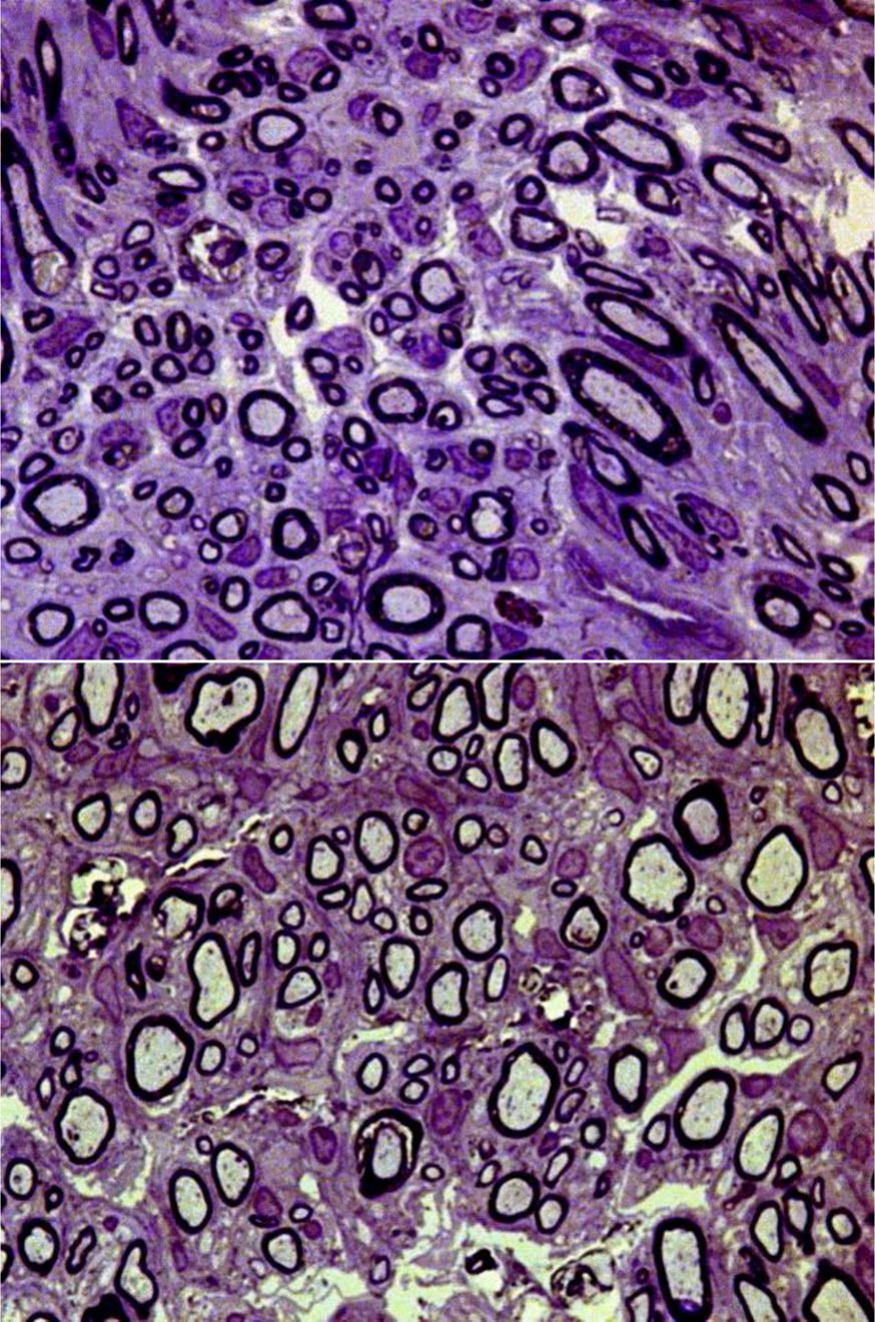
In the image above, a semithin cross-section of the **distal segment** of the sciatic nerve from a rat in **group C**, stained with toluidine blue and viewed at ×1000 magnification. In the imagen below, a semithin cross-section of the **middle segment** of the sciatic nerve from the same animal, stained with toluidine blue and viewed at 1000 × magnification

**Fig. 9:**
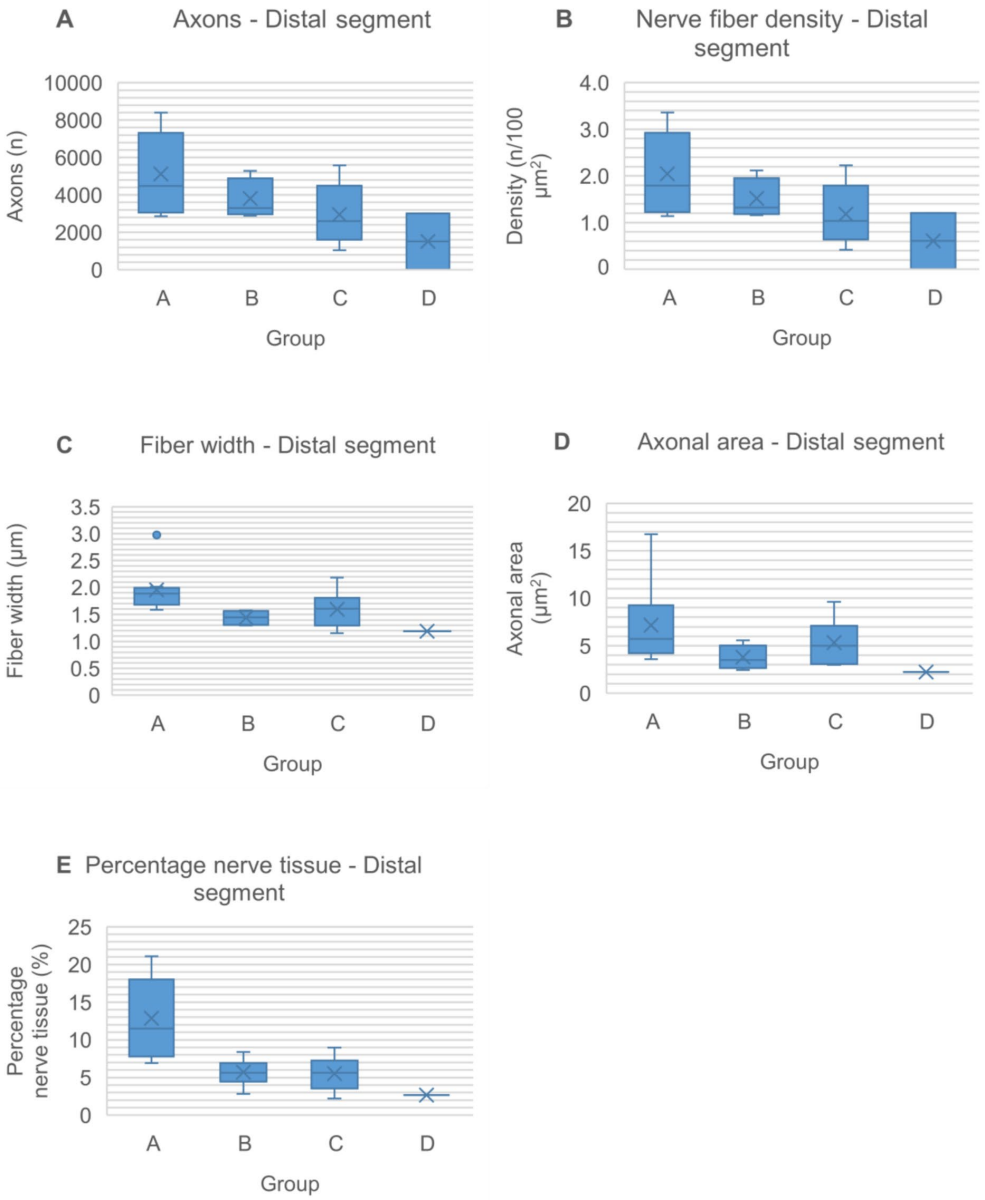
Histomorphometric findings reveal axonal regeneration through fresh nerve allografts, lyophilized decellularized nerve allografts, fresh decellularized nerve allografts and silicone tube 20 weeks postoperatively. Box-plots represent the data as mean ± standard deviation at the distal segment of the nerve at each experimental group (**A**–**D**) for different variables: axon count, nerve fiber density, fiber width, axonal area and percentage nerve tissue

**Fig. 10:**
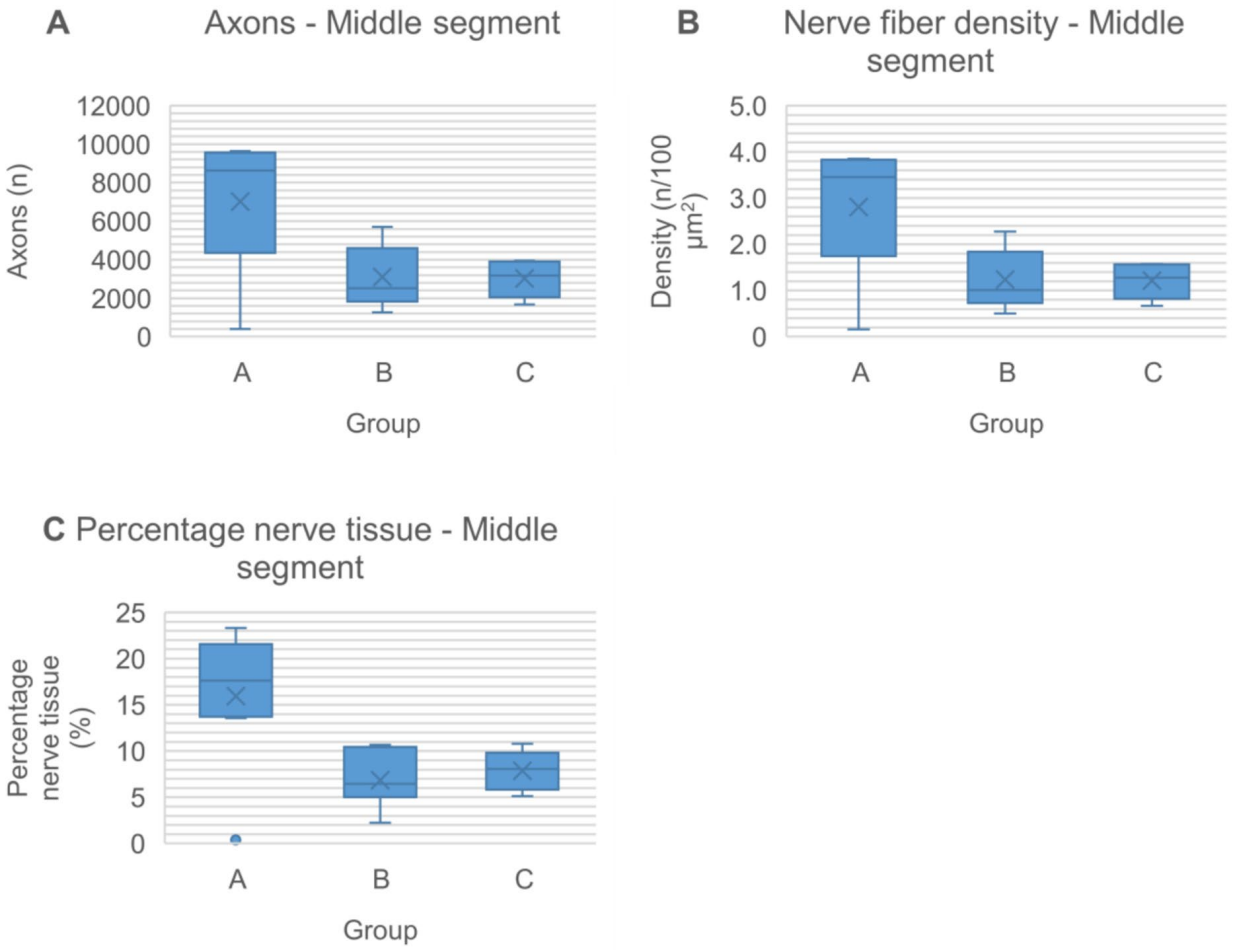
Histomorphometric findings reveal axonal regeneration through fresh nerve autografts, lyophilized decellularized nerve allografts fresh decellularized nerve allografts, and silicone tube 20 weeks postoperatively. Box-plots represent the data as mean ± standard deviation at the middle segment of the nerve at each experimental group (**A**–**D**) for different variables: axon count, nerve fiber density and percentage nerve tissue

At 20 weeks postoperatively, all nerve grafts maintained continuity, with no signs of degeneration, rejection or neuroma formation (Figs. 6, 7 and 8). The total number of myelinated axons did not significantly differ between nerve autografts and lyophilized or fresh acellular allografts. However, differences were observed in the number of axons in the middle segment among groups, though not statistically significant after adjustment (Figs. 9A and 10A) (Tables 5 and 6). There was not any sign of axonal growth through the silicone tube.

**Table 5:** Normalized axonal count at the distal segment of the repaired sciatic nerve for each experimental group (A–D)

Group (n)	Median	Minimum	Maximum	Mean	Standard deviation
A (8)	4483.00	2854.00	8400.00	5113.12	2213.82
B (7)	3301.50	2888.00	5290.00	3800.89	984.05
C (6)	2593.00	1038.00	5576.00	2952.88	1646.56
D (2)	1511.88	14.00	3009.00	1511.88	2117.61

A: nerve autograft. B: lyophilized decellularized nerve allografts, C: fresh decellularized nerve allografts, D: silicone tube. The n stands for number of animals in the sample

**Table 6:** Normalized axonal count at the middle segment of the repaired sciatic nerve for each experimental group (A–D)

Group (n)	Median	Minimum	Maximum	Mean	Standard deviation
A (8)	8628.00	409.00	9636.00	7017.62	3375.08
B (7)	2526.00	1256.00	5698.00	3099.43	1569.71
C (5)	3184.00	1670.00	3936.00	3025.60	971.64
D (0)					

A: nerve autografts, B: lyophilized decellularized nerve allografts, C: fresh decellularized nerve allografts, D: silicone tube. The n stands for number of animals in the sample

Comparing nerve fiber density between the nerve autograft and acellular allograft groups did not revealed significant differences. In the distal segment, the autograft group (group A) exhibited the highest fiber density, followed by the lyophilized acellular allograft group (group B) and the fresh acellular allograft group (group C) (Table 7). Similarly, in the middle segment, fresh acellular allografts showed the highest fiber density after group A (Table 8). However, these differences did not achieve statistical significance following the Holm-Bonferroni adjustment (Fig. 9B and 10B).

**Table 7:** Nerve fiber density (number of axons/100 µm^2^) at the distal segment of the nerves in each group

Group (n)	Median	Minimum	Maximum	Mean	Standard deviation
A (8)	1.79	1.14	3.36	2.05	0.89
B (7)	1.32	1.16	2.12	1.52	0.39
C (6)	1.04	0.42	2.23	1.18	0.66
D (2)	0.60	0.01	1.20	0.60	0.85

The n represents the sample size in each group for this variable

**Table 8:** Nerve fiber density (number of axons/100 µm^2^) at the middle segment of the nerves in each group

Group (n)	Median	Minimum	Maximum	Mean	Standard deviation
A (8)	3.45	0.16	3.85	2.81	1.35
B (7)	1.01	0.50	2.28	1.24	0.63
C (5)	1.27	0.67	1.57	1.21	0.39
D (0)					

The n represents the sample size in each group for this variable

Although fiber width tended to be greater in the middle segment compared to the distal segment in all groups, no significant differences in axon diameter were observed between any groups in either segment (Fig. 9C) (Tables 9 and 10).

**Table 9:** Nerve fiber width (µm) at the distal segment of the nerves in each group

Group (n)	Median	Minimum	Maximum	Mean	Standard deviation
A (8)	1.88	1.58	2.98	1.96	0.44
B (7)	1.45	1.30	1.57	1.44	0.12
C (6)	1.60	1.16	2.18	1.59	0.35
D (0)					

The n represents the sample size in each group for this variable

**Table 10:** Nerve fiber width (µm) at the middle segment of the nerves in each group

Group (n)	Median	Minimum	Maximum	Mean	Standard deviation
A (8)	1.97	1.36	2.24	1.91	0.27
B (7)	1.67	1.56	2.52	1.86	0.39
C (5)	1.95	1.75	2.16	1.96	0.16
D (0)					

The n represents the sample size in each group for this variable

In assessing axonal characteristics, particularly axonal area and myelin sheath thickness (measured in µm), no significant differences were found between groups in either the distal or middle segments of the sciatic nerve. Despite the proximity of the p-value to significance in the median comparison test for axonal area in the distal segment, no statistically significant distinctions were evident (Fig. 9D) (Tables 11 and 12). Moreover, substantial variability in myelin sheath thickness across measured axons was observed, highlighting the diversity within the dataset (Tables 13 and 14).

**Table 11:** Axonal area (µm^2^) at the distal segment of the nerves in each group

Group (n)	Median	Minimum	Maximum	Mean	Standard deviation
A (8)	5.73	3.60	16.74	7.16	4.38
B (7)	3.50	2.44	5.57	3.80	1.16
C (6)	4.99	2.99	9.62	5.32	2.47
D (0)					

The n represents the sample size in each group for this variable

**Table 12:** Axonal area (µm^2^) at the middle segment of the nerves in each group

Group (n)	Median	Minimum	Maximum	Mean	Standard deviation
A (8)	5.40	2.30	11.53	5.69	2.65
B (7)	4.86	3.05	10.79	6.19	3.06
C (5)	6.68	5.61	7.66	6.64	0.75
D (0)					

The n represents the sample size in each group for this variable

**Table 13:** Myelin sheath thickness (µm) at the distal segment of the nerves in each group

Group (n)	Median	Minimum	Maximum	Mean	Standard deviation
A (8)	5.23	0.75	17.08	6.30	5.68
B (7)	3.39	0.76	10.45	4.58	3.62
C (6)	6.60	0.80	12.71	6.40	4.20
D (0)					

The n represents the sample size in each group for this variable

**Table 14:** Myelin sheath thickness (µm) at the middle segment of the nerves in each group

Group (n)	Median	Minimum	Maximum	Mean	Standard deviation
A (8)	2.10	0.43	13.86	3.16	4.42
B (7)	4.37	0.70	12.72	4.82	4.34
C (5)	2.78	0.76	3.27	2.10	1.23
D (0)					

The n represents the sample size in each group for this variable

Evaluation of nerve tissue percentage in nerve defects treated with different graft types demonstrated higher percentages in the distal and middle nerve segments of those repaired with nerve autografts (group A) compared to lyophilized acellular allografts (group B) and fresh acellular nerve allografts (group C). Although significant differences were initially detected using the Kruskal–Wallis median comparison test, in the distal segment (*p* value = 0.006) and middle segment (*p* value = 0.018), these distinctions were no longer significant post Holm-Bonferroni adjustment (Figs. 9E and 10C) (Tables 15 and 16).

**Table 15:** Percentage nerve tissue (%) at the distal segment of the nerves in each group

Group (n)	Median	Minimum	Maximum	Mean	Standard deviation
A (8)	11.49	6.91	21.07	12.87	5.43
B (7)	5.61	2.82	8.40	5.69	1.86
C (6)	5.62	2.21	8.97	5.52	2.40
D (0)					

The n represents the sample size in each group for this variable

**Table 16:** Percentage nerve tissue (%) at the middle segment of the nerves in each group

Group (n)	Median	Minimum	Maximum	Mean	Standard deviation
A (8)	17.62	0.38	23.32	15.96	7.19
B (7)	6.46	2.22	10.67	6.87	2.96
C (5)	8.05	5.11	10.80	7.87	2.18
D (0)					

The n represents the sample size in each group for this variable

Furthermore, the regeneration index, calculated as the ratio of axon count in the distal segment to that in the proximal segment, varied across all groups and included instances of a regeneration index greater than 1 and others lower than 1 within the same experimental group. Subsequent hypothesis testing revealed no significant differences between groups (*p* value = 0.512) (Table 17).

**Table 17:** Regeneration index of the sciatic nerve in each group

Group (n)	Median	Minimum	Maximum	Mean	Standard deviation
A (8)	0.80	0.48	2.35	1.00	0.60
B (6)	1.07	0.63	3.23	1.52	1.04
C (6)	0.92	0.36	2.18	1.06	0.63
D (2)	0.45	0.00	0.89	0.45	0.62

The n represents the sample size in each group for this variable

### Muscle histomorphometry

**Fig. 11:**
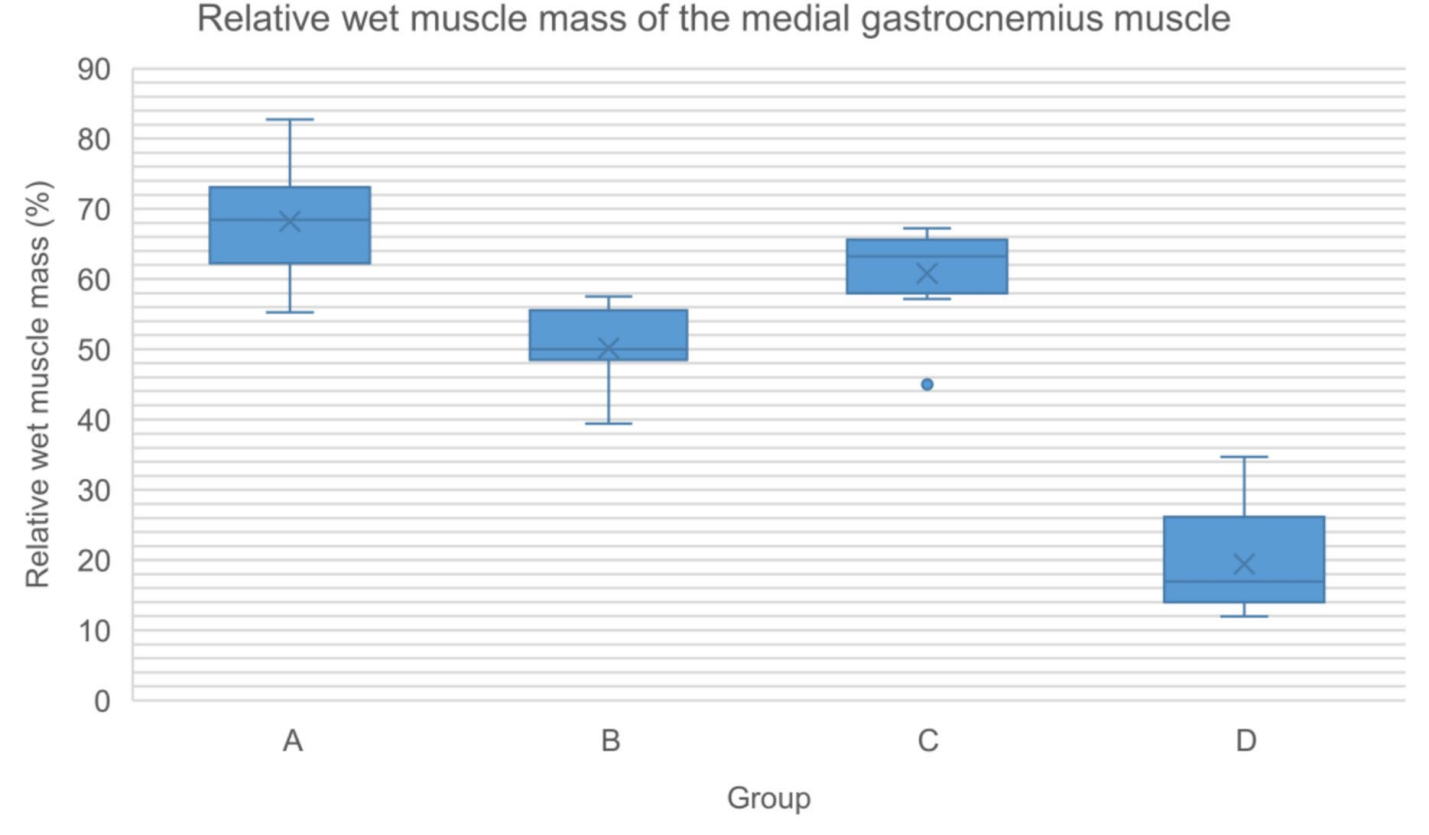
Relative wet muscle mass of the medial gastrocnemius muscle (%)

**Fig. 12:**
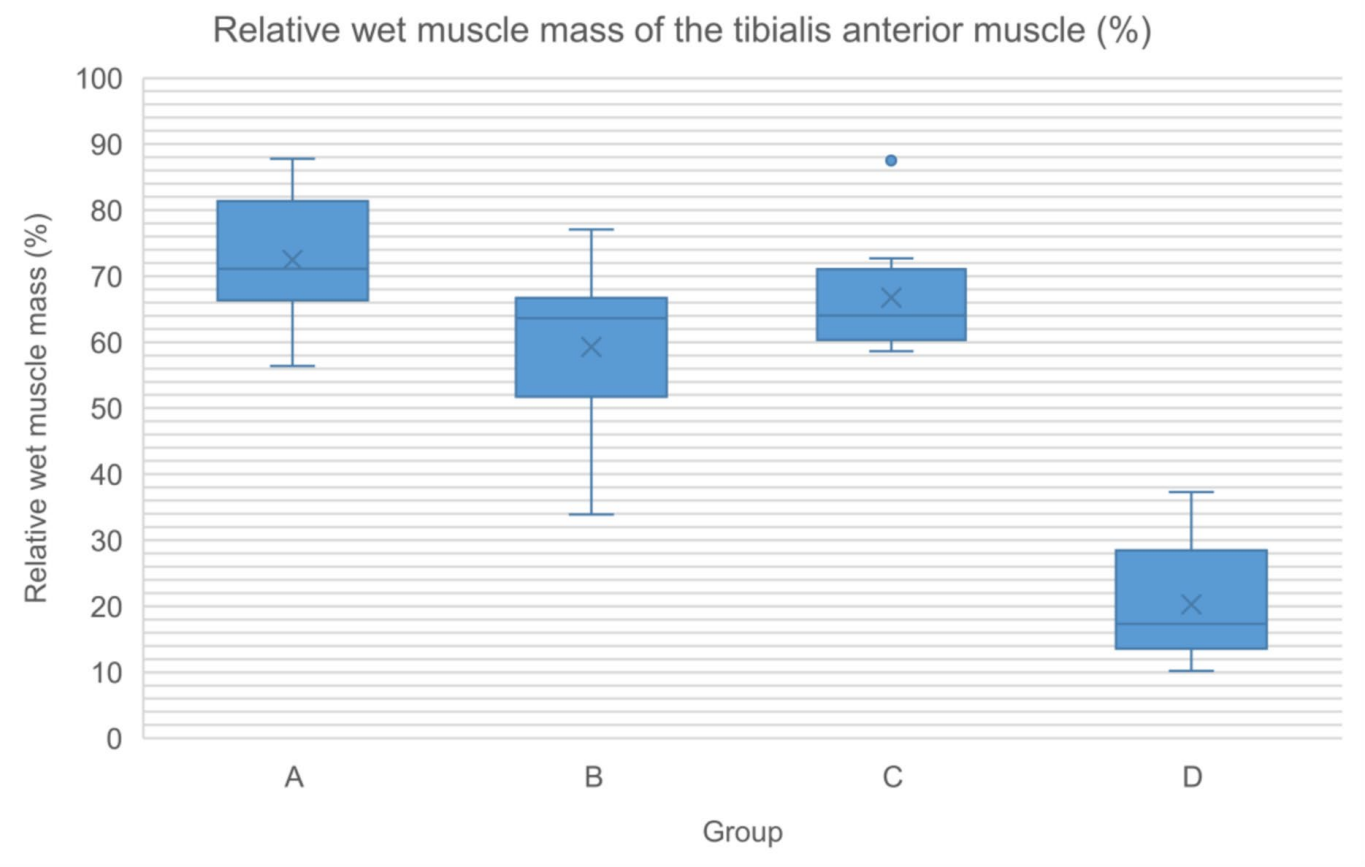
Relative wet muscle mass of the tibialis anterior muscle (%)

Relative muscle mass analysis revealed significant differences in the medial gastrocnemius muscle mass between groups, with the highest values observed in groups A and C, followed by B, and the lowest in group D (Table 18, Fig. 11). No significant differences were found in the tibialis anterior muscle mass between groups (Table 19, Fig. 12).

**Table 18:** Relative wet muscle mass of the medial gastrocnemius muscle (%)

Group (n)	Median	Minimum	Maximum	Mean	Standard deviation
A (8)	68.42	55.22	82.76	68.20	8.47
B (7)	50.00	39.39	57.53	58.14	5.83
C (8)	63.22	45.00	67.24	60.81	7.14
D (5)	16.95	11.94	34.69	19.44	8.80

**Table 19:** Relative wet muscle mass of the tibialis anterior muscle (%)

Group (n)	Median	Minimum	Maximum	Mean	Standard deviation
A (8)	71.10	56.36	87.76	72.44	9.92
B (7)	63.64	33.90	77.05	59.25	13.71
C (8)	64.02	58.62	87.50	66.77	9.45
D (5)	17.31	10.20	37.29	20.28	10.14

**Fig. 13:**
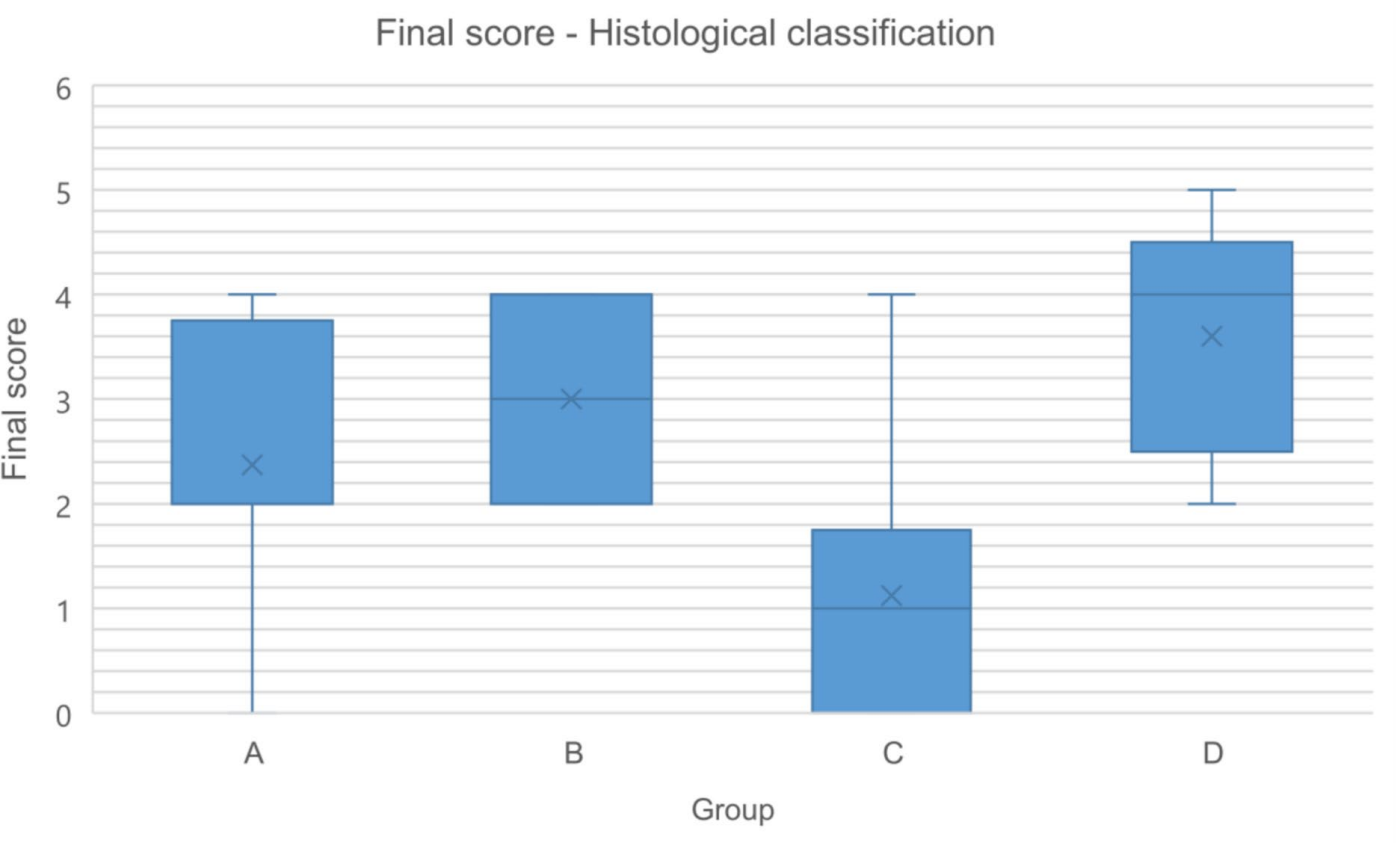
Final score (0–4) at the histological classification of the left medial gastrocnemius muscle for each group

**Fig. 14:**
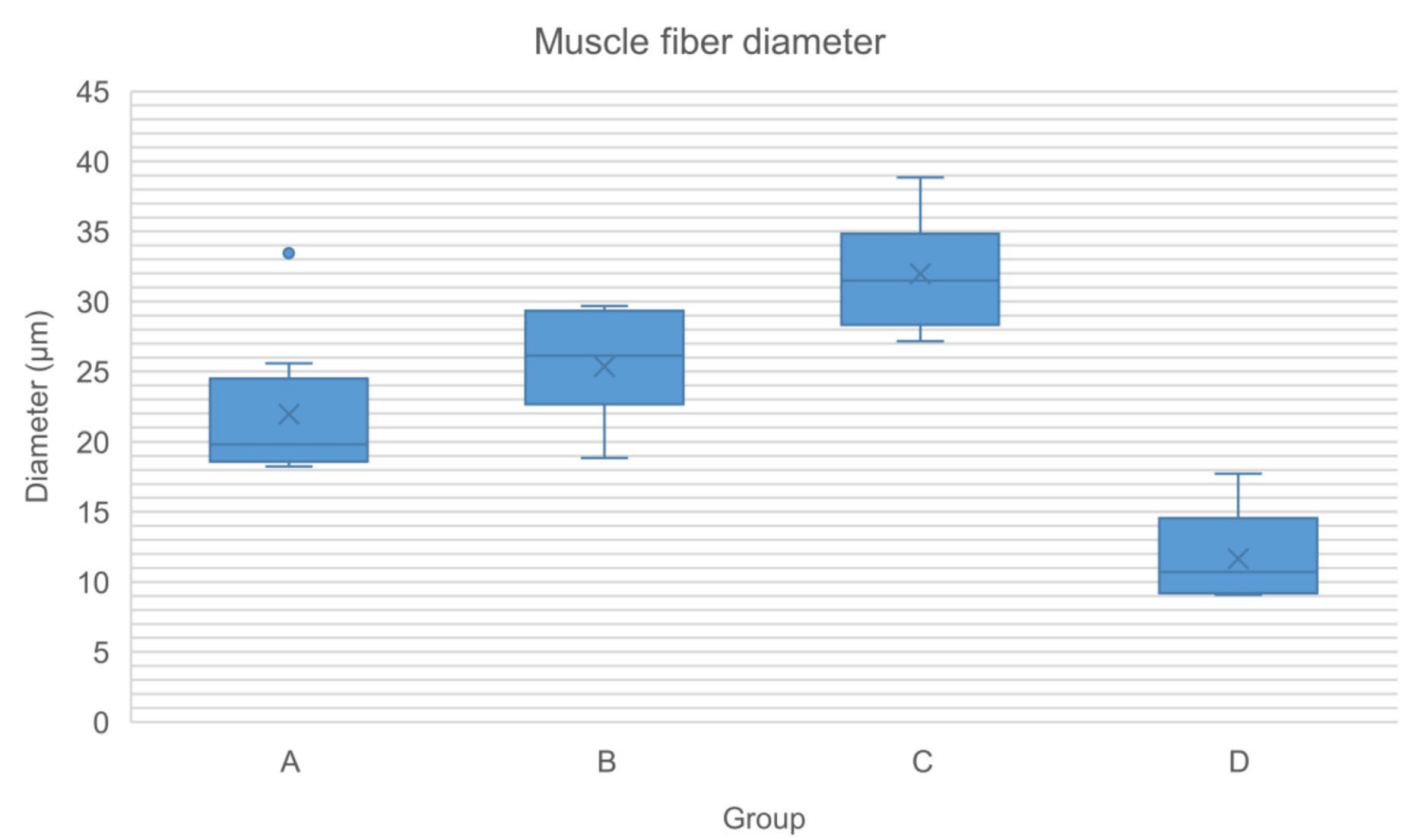
Muscle fiber diameter (μm) of the reinnervated medial gastrocnemius muscle in each group

Histological assessment showed no significant differences in the final score of the histological classification of the medial gastrocnemius muscle (Tables 20 and 21, Fig. 13). However, within this score system, group C showed significantly larger muscle fiber diameters (mean = 31.48 µm) compared to groups A and B, that displayed muscle fiber diameters of 19.82 µm and 26.12 µm, respectively (Table 22 and Fig. 14).

**Table 20:** Relative frequencies (%) and absolute frequencies (in parentheses) of variables comprising the histological classification of the medial gastrocnemius muscle

Variable		A	B	C	D
Muscle fiber atrophy	0	12.5 (1)	0.0 (0)	50.0 (4)	0.0 (0)
	1	87.5 (7)	100.0 (7)	50.0 (4)	20.0 (1)*
	2	0.0 (0)	0.0 (0)	0.0 (0)	80.0 (4)*
Central nucleii	0	12.5 (1)	42.9 (3)	75.0 (6)	20.0 (1)
	1	87.5 (7)	57.1 (4)	25.0 (2)	80.0 (4)
	2	0.0 (0)	0.0 (0)	0.0 (0)	0.0 (0)
Collagen infiltration	0	62.5 (5)	0.0 (0)	75.0 (6)	20.0 (1)
	1	37.5 (3)	100.0 (7)	25.0 (2)	80.0 (4)
	2	0.0 (0)	0.0 (0)	0.0 (0)	0.0 (0)
Fatty degeneration	0	75.0 (6)	57.1 (4)	87.5 (7)	80.0 (4)
	1	25.0 (2)	42.9 (3)	12.5 (1)	20.0 (1)
	2	0.0 (0)	0.0 (0)	0.0 (0)	0.0 (0)

0 = no affected muscle tissue. 1 =  ≤ 50% affected muscle tissue. 2 =  > 50% affected muscle tissue. Statistically significant differences are denoted with an asterisk

**Table 21:** Final score (0–4) at the histological classification of the left medial gastrocnemius muscle for each group

Group (n)	Median	Minimum	Maximum	Mean	Standard deviation
A (8)	2.00	0.00	4.00	2.38	1.30
B (7)	3.00	2.00	4.00	3.00	0.82
C (8)	1.00	0.00	4.00	1.12	1.36
D (5)	4.00	2.00	5.00	3.60	1.14

**Table 22:** Muscle fiber diameter (μm) of the reinnervated medial gastrocnemius muscle in each group

Group (n)	Median	Minimum	Maximum	Mean	Standard deviation
A (8)	19.82	18.24	33.44	21.94	5.22
B (7)	26.12	18.86	29.67	25.34	3.95
C (8)	31.48*	27.15	38.84	31.98	4.03
D (5)	10.72*	9.08	17.72	11.65	3.53

## Discussion

Nerve morphological results indicate that both lyophilized and fresh-preserved decellularized allografts facilitate axonal growth comparably to autografts, outperforming empty silicone tubes. The absence of regeneration in the silicone conduit group confirms its role as a negative control (Brenner et al. 2008; Lundborg et al. 1982; Williams et al. 1983).

One of the key strengths of this study lies in the meticulous effort to minimize biases when quantifying nerve morphological variables. In this experiment, each image underwent random sampling to ensure equitable representation across all fields, adhering to the "equal opportunity" rule (Geuna et al. 2004). To mitigate overestimation and edge effects, incomplete fibers touching lower and right edges were excluded, and manual adjustment of counts was permitted to rectify any erroneous inclusions or exclusions.

Previous literature suggests examining a minimum of 10% of the sampled area to mitigate selection bias, a criterion met in our study, with most segments allowing analysis of at least 25% of the total sampled area (Hunter et al. 2007; Prodanov and Feirabend 2007; Torch et al. 1989). It is worth noting that Brenner highlighted the risk of type II errors in rodent models and emphasized the importance of selecting appropriate endpoints for histological and functional nerve analysis based on defect type and size (Brenner et al. 2008). Our chosen 20-week endpoint strikes a balance between optimal axonal regeneration and functional recovery for a 14 mm defect.

Our findings align with similar axon counts reported in corresponding groups from previous studies. Moore et al. demonstrated that decellularized allografts treated with detergents achieved a comparable number of axons in the distal segment to autografts and a higher number compared to decellularized allografts processed by AxoGen® (Avance nerve grafts, AxoGen, Inc., Alachua, Florida) or cold processing methods (Moore et al. 2011). Even though other authors reported higher axonal counts, these differences could be potentially influenced by factors like animal weight and defect size (Tang et al. 2013; Whitlock et al. 2009).

Regenerated axons across decellularized nerve allografts achieved target muscle reinnervation. Both medial gastrocnemius and tibialis anterior relative muscle mass recovered to levels comparable to those animals treated with autografts and performed significantly better than those treated with silicone tubes (negative control group).

Fresh-preserved acellular allografts demonstrated the highest scores in detailed muscle histological assessment, followed by autografts and lyophilized acellular allografts, with the negative control group exhibiting the lowest scores. Although overall scores did not reveal statistical significance, differences in muscle fiber atrophy and diameter were evident. Animals treated with fresh acellular allografts had significantly larger fiber diameters compared to autograft or lyophilized acellular allograft-treated animals. Muscle atrophy represented as the shrinking of the muscle fiber, preceding collagen deposition and predisposing to muscle fibrosis is pertinent to our findings, where muscle atrophy affected more animals within each group than collagen deposition, which typically initiates around three months post-denervation (Aird & Naffziger 1953).

Consistent results were reported by Tang et al. and Moore et al., where no significant differences were observed between experimental groups in terms of muscle weight when compared to muscle reinnervated through autografts (Moore et al. 2011; Tang et al. 2013).

Finally, the project focused on evaluating the functional recovery after nerve repair at week 20 after surgery. Initial examination of functional nerve regeneration revealed the absence of statistically significant differences in assessed functional indexes or the Rotarod test (Whitlock et al. 2009). Prior research advised against the confounding factors that may mask underlying differences between groups such as fixed muscle contractures and aberrant reinnervation, which lead to discrepancies between functional results and histological and morphological data (Dellon and Mackinnon 1989; Hare et al. 1992; Weber et al. 1993). This abnormal axonal growth is aggravated by the section-type injury model used in this experiment, which does not preserve the epineurum, contrary to crush-type injury models. This troubles proper fiber growth and results in altered functional indexes (Dellon and Mackinnon 1989; Wang et al. 2018; Weber et al. 1993). Another phenomenon hiding functional differences between groups is polyneural innervation. This is a compensatory mechanism where denervation triggers collateral axonal growth and motor unit multiplication (Ijkema-Paassen et al. 2002; Valero-Cabré and Navarro 2002; Wood et al. 2011). Even though many of the papers on sciatic nerve regeneration through allografts based their functional results on functional indexes, it is also one of the limitations of the study. Employing retrograde labelling of motor neurons and more precise functional assessments could enhance understanding, albeit at increased cost and duration (Vleggeert-Lankamp 2007; Wood et al. 2011, 2014).

Our study demonstrates that acellular allografts support axonal regeneration in 14 mm mixed defects comparably to autografts and surpass silicone tubes. Although histological differences were non-significant, accurate muscle reinervation assessed through muscle histology appeared more promising in animals treated with fresh-preserved acellular allografts. This finding suggests that the preservation method may influence muscle recovery. The method of decellularization of allografts is the same in both cases and they only differ in the last step, related to their preservation method. This last step would have consequences evidenced in the differences observed at the muscular level.

One drawback of this study is the absence of a comparison between detergent-processed acellular allografts and the currently available AxoGen® allografts. Our study did not aim to directly compare our allografts with Axogen’s products, as Moore et al. (2011) previously conducted such a comparison and found no superiority of Axogen’s detergent-processed allografts. Moreover, incorporating such a comparison would have required additional animals and shifted the focus away from our primary objective: validating our model in vivo. Additionally, obtaining Axogen allografts for experimental use is both difficult and costly (Moore et al. 2011).

With the forward-looking objective of translating these findings into clinical applications in humans, it is imperative to optimize all neural processing mechanisms accordingly. Our developed method facilitates axonal regeneration and functional recovery comparably, if not superiorly, to autografts in rats. The decellularization protocol is technically simple and straightforward. It involves a 24-h incubation followed by continuous agitation for three days using 0.1% sodium dodecyl sulfate (SDS) and 1× phosphate-buffered saline (PBS). These reagents are readily available in standard laboratories, do not require specialized equipment, and are cost-effective. Additionally the protocol can be tailored to suit the nature of human nerves. In fact, the pilot study for clinical application commenced shortly after the presentation of these findings in 2023 and has now been completed with a cohort of 10 patients. Subsequently, decellularized nerve allografts have been implanted in up to 30 patients, demonstrating satisfactory and comparable clinical outcomes. Leveraging the Tissue Bank in our region, fresh acellular nerve allografts from human donors—processed through a similar decellularization protocol as outlined in this study—are already available. Consequently, the allografts developed in this study, now authorized by the Organización Nacional de Trasplantes (ONT), can be distributed within Spain and theoretically throughout the European Union However, such cross-border distribution is uncommon, as tissue bank activities in Spain are generally localized. This provides the foundation for developing a lyophilized alternative, which would result in a highly valuable biomaterial, reducing harvesting and manufacturing costs, while also addressing stock and storage challenges. Human acellular nerves could allow us replacing collagen conduits and autografts for treating certain injuries in clinical practice.

## Data Availability

No datasets were generated or analysed during the current study.

## References

[CR1] Aird RB, Naffziger HC (1953) The pathology of human striated muscle following denervation. J Neurosurg 10(3):216–227. 10.3171/jns.1953.10.3.0216 13053251 10.3171/jns.1953.10.3.0216

[CR2] Bain JR (1998) Peripheral nerve allografting: review of the literature with relevance to composite tissue transplantation. Transplant Proc 30(6):2762–2767 9745562 10.1016/s0041-1345(98)00804-5

[CR3] Brenner MJ, Moradzadeh A, Myckatyn TM, Tung THH, Mendez AB, Hunter DA, Mackinnon SE (2008) Role of timing in assessment of nerve regeneration. Microsurgery 28(4):265–272. 10.1002/micr.20483 18381659 10.1002/micr.20483 PMC4027974

[CR4] de Medinaceli L, Freed WJ, Wyatt RJ (1982) An index of the functional condition of rat sciatic nerve based on measurements made from walking tracks. Exp Neurol 77(3):634–643 7117467 10.1016/0014-4886(82)90234-5

[CR5] Dellon AL, Mackinnon SE (1989) Sciatic nerve regeneration in the rat. Validity of walking track assessment in the presence of chronic contractures. Microsurgery 10(3):220–225. 10.1002/micr.1920100316 2796718 10.1002/micr.1920100316

[CR6] Dumanian GA, Potter BK, Mioton LM, Ko JH, Cheesborough JE, Souza JM, Ertl WJ, Tintle SM, Nanos GP, Valerio IL, Kuiken TA, Apkarian AV, Porter K, Jordan SW (2019) Targeted muscle reinnervation treats neuroma and phantom pain in major limb amputees: a randomized clinical trial. Ann Surg 270(2):238–246. 10.1097/SLA.0000000000003088 30371518 10.1097/SLA.0000000000003088

[CR7] Geuna S, Gigo-Benato D, Rodrigues A de C (2004) On sampling and sampling errors in histomorphometry of peripheral nerve fibers. Microsurgery 24(1):72–76. 10.1002/micr.10199 10.1002/micr.10199 14748030

[CR8] Gordon T, Chan KM, Sulaiman OAR, Udina E, Amirjani N, Brushart TM (2009) Accelerating axon growth to overcome limitations in functional recovery after peripheral nerve injury. Neurosurgery 65(4 Suppl):A132-144. 10.1227/01.NEU.0000335650.09473.D3 19927058 10.1227/01.NEU.0000335650.09473.D3

[CR9] Hamm RJ, Pike BR, O’Dell DM, Lyeth BG, Jenkins LW (1994) The rotarod test: an evaluation of its effectiveness in assessing motor deficits following traumatic brain injury. J Neurotrauma 11(2):187–196. 10.1089/neu.1994.11.187 7932797 10.1089/neu.1994.11.187

[CR10] Hare G, Evans P, Mackinnon S, Best T, Bain J, Szalai J, Hunter D (1992) Walking track analysis—A long-term assessment of peripheral-nerve recovery. Plast Reconstr Surg 89(2):251–258. 10.1097/00006534-199202000-00009 1732892

[CR11] Hong T, Wood I, Hunter DA, Yan Y, Mackinnon SE, Wood MD, Moore AM (2019) Neuroma management: capping nerve injuries with an acellular nerve allograft can limit axon regeneration. Hand (New York, NY). 10.1177/1558944719849115 10.1177/1558944719849115 PMC8041431 31137979

[CR12] Hunter DA, Moradzadeh A, Whitlock EL, Brenner MJ, Myckatyn TM, Wei CH, Tung THH, Mackinnon SE (2007) Binary imaging analysis for comprehensive quantitative histomorphometry of peripheral nerve. J Neurosci Methods 166(1):116–124. 10.1016/j.jneumeth.2007.06.018 17675163 10.1016/j.jneumeth.2007.06.018 PMC2587177

[CR13] Ijkema-Paassen J, Meek MF, Gramsbergen A (2002) Reinnervation of muscles after transection of the sciatic nerve in adult rats. Muscle Nerve 25(6):891–897. 10.1002/mus.10130 12115979 10.1002/mus.10130

[CR14] Isaacs J (2013) Major peripheral nerve injuries. Hand Clin 29(3):371–382. 10.1016/j.hcl.2013.04.006 23895717 10.1016/j.hcl.2013.04.006

[CR15] Ives GC, Kung TA, Nghiem BT, Ursu DC, Brown DL, Cederna PS, Kemp SWP (2017) Current state of the surgical treatment of terminal neuromas. Neurosurgery. 10.1093/neuros/nyx500 10.1093/neuros/nyx500 29053875

[CR16] Johnson PC, Duhamel RC, Meezan E, Brendel K (1982) Preparation of cell-free extracellular matrix from human peripheral nerve. Muscle Nerve 5(4):335–344. 10.1002/mus.880050410 7099200 10.1002/mus.880050410

[CR17] Kubiak CA, Kung TA, Brown DL, Cederna PS, Kemp SWP (2018) State-of-the-art techniques in treating peripheral nerve injury. Plast Reconstr Surg 141(3):702. 10.1097/PRS.0000000000004121 29140901 10.1097/PRS.0000000000004121

[CR18] Lundborg G, Dahlin LB, Danielsen N, Gelberman RH, Longo FM, Powell HC, Varon S (1982) Nerve regeneration in silicone chambers: influence of gap length and of distal stump components. Exp Neurol 76(2):361–375. 10.1016/0014-4886(82)90215-1 7095058 10.1016/0014-4886(82)90215-1

[CR19] Martins RS, Siqueira MG, da Silva CF, Plese JPP (2006) Correlation between parameters of electrophysiological, histomorphometric and sciatic functional index evaluations after rat sciatic nerve repair. Arq Neuropsiquiatr 64(3B):750–756 17057880 10.1590/s0004-282x2006000500010

[CR20] Moore AM, MacEwan M, Santosa KB, Chenard KE, Ray WZ, Hunter DA, Mackinnon SE, Johnson PJ (2011) Acellular nerve allografts in peripheral nerve regeneration: a comparative study. Muscle Nerve 44(2):221–234. 10.1002/mus.22033 21660979 10.1002/mus.22033 PMC3136642

[CR21] Noble J, Munro CA, Prasad VS, Midha R (1998) Analysis of upper and lower extremity peripheral nerve injuries in a population of patients with multiple injuries. J Trauma 45(1):116–122 9680023 10.1097/00005373-199807000-00025

[CR22] Pan D, Mackinnon SE, Wood MD (2020) Advances in the repair of segmental nerve injuries and trends in reconstruction. Muscle Nerve 61(6):726–739. 10.1002/mus.26797 31883129 10.1002/mus.26797 PMC7230025

[CR23] Prodanov D, Feirabend HKP (2007) Morphometric analysis of the fiber populations of the rat sciatic nerve, its spinal roots, and its major branches. J Comp Neurol 503(1):85–100. 10.1002/cne.21375 17480027 10.1002/cne.21375

[CR24] Robinson LR (2000) Traumatic injury to peripheral nerves. Muscle Nerve 23(6):863–873 10842261 10.1002/(sici)1097-4598(200006)23:6<863::aid-mus4>3.0.co;2-0

[CR25] Sachanandani NF, Pothula A, Tung TH (2014) Nerve gaps. Plast Reconstr Surg 133(2):313. 10.1097/01.prs.0000436856.55398.0f 24150118 10.1097/01.prs.0000436856.55398.0f

[CR26] Safa B, Buncke G (2016) Autograft substitutes: conduits and processed nerve allografts. Hand Clin 32(2):127–140. 10.1016/j.hcl.2015.12.012 27094886 10.1016/j.hcl.2015.12.012

[CR27] Saheb-Al-Zamani M, Yan Y, Farber SJ, Hunter DA, Newton P, Wood MD, Stewart SA, Johnson PJ, Mackinnon SE (2013) Limited regeneration in long acellular nerve allografts is associated with increased Schwann cell senescence. Exp Neurol 247:165–177. 10.1016/j.expneurol.2013.04.011 23644284 10.1016/j.expneurol.2013.04.011 PMC3863361

[CR28] Schindelin J, Arganda-Carreras I, Frise E, Kaynig V, Longair M, Pietzsch T, Preibisch S, Rueden C, Saalfeld S, Schmid B, Tinevez J-Y, White DJ, Hartenstein V, Eliceiri K, Tomancak P, Cardona A (2012) Fiji: an open-source platform for biological-image analysis. Nat Methods 9(7):Article 7. 10.1038/nmeth.2019 10.1038/nmeth.2019 PMC3855844 22743772

[CR29] Schneider CA, Rasband WS, Eliceiri KW (2012) NIH image to ImageJ: 25 years of image analysis. Nat Methods 9(7):671–675. 10.1038/nmeth.2089 22930834 10.1038/nmeth.2089 PMC5554542

[CR30] Sondell M, Lundborg G, Kanje M (1998) Regeneration of the rat sciatic nerve into allografts made acellular through chemical extraction. Brain Res 795(1–2):44–54 9622591 10.1016/s0006-8993(98)00251-0

[CR31] Szynkaruk M, Kemp SWP, Wood MD, Gordon T, Borschel GH (2013) Experimental and clinical evidence for use of decellularized nerve allografts in peripheral nerve gap reconstruction. Tissue Eng B Rev 19(1):83–96. 10.1089/ten.TEB.2012.0275 10.1089/ten.TEB.2012.0275 22924762

[CR32] Tang P, Kilic A, Konopka G, Regalbuto R, Akelina Y, Gardner T (2013) Histologic and functional outcomes of nerve defects treated with acellular allograft versus cabled autograft in a rat model. Microsurgery 33(6):460–467. 10.1002/micr.22102 23861174 10.1002/micr.22102

[CR33] Torch S, Usson Y, Saxod R (1989) Automated morphometric study of human peripheral nerves by image analysis. Pathol Res Pract 185(5):567–571. 10.1016/S0344-0338(89)80195-5 2626366 10.1016/S0344-0338(89)80195-5

[CR34] Valero-Cabré A, Navarro X (2002) Functional impact of axonal misdirection after peripheral nerve injuries followed by graft or tube repair. J Neurotrauma 19(11):1475–1485. 10.1089/089771502320914705 12490012 10.1089/089771502320914705

[CR35] Vleggeert-Lankamp CLAM (2007) The role of evaluation methods in the assessment of peripheral nerve regeneration through synthetic conduits: a systematic review. Laboratory investigation. J Neurosurg 107(6):1168–1189. 10.3171/JNS-07/12/1168 18077955 10.3171/JNS-07/12/1168

[CR36] wamsleyk (2016, mayo 12) Strategies of peripheral nerve surgery. Surgical Education. https://surgicaleducation.wustl.edu/strategies-of-peripheral-nerve-surgery/

[CR37] Wang T, Ito A, Aoyama T, Nakahara R, Nakahata A, Ji X, Zhang J, Kawai H, Kuroki H (2018) Functional evaluation outcomes correlate with histomorphometric changes in the rat sciatic nerve crush injury model: a comparison between sciatic functional index and kinematic analysis. PLoS ONE 13(12):e0208985. 10.1371/journal.pone.0208985 30540822 10.1371/journal.pone.0208985 PMC6291147

[CR38] Weber RA, Proctor WH, Warner MR, Verheyden CN (1993) Autotomy and the sciatic functional index. Microsurgery 14(5):323–327. 10.1002/micr.1920140507 8332052 10.1002/micr.1920140507

[CR39] Whitlock EL, Tuffaha SH, Luciano JP, Yan Y, Hunter DA, Magill CK, Moore AM, Tong AY, Mackinnon SE, Borschel GH (2009) Processed allografts and type I collagen conduits for repair of peripheral nerve gaps. Muscle Nerve 39(6):787–799. 10.1002/mus.21220 19291791 10.1002/mus.21220

[CR40] Williams LR, Longo FM, Powell HC, Lundborg G, Varon S (1983) Spatial-temporal progress of peripheral nerve regeneration within a silicone chamber: parameters for a bioassay. J Comp Neurol 218(4):460–470. 10.1002/cne.902180409 6619324 10.1002/cne.902180409

[CR41] Wood MD, Mackinnon SE (2015) Pathways regulating modality-specific axonal regeneration in peripheral nerve. Exp Neurol 265:171–175. 10.1016/j.expneurol.2015.02.001 25681572 10.1016/j.expneurol.2015.02.001 PMC4399493

[CR42] Wood MD, Kemp SWP, Weber C, Borschel GH, Gordon T (2011) Outcome measures of peripheral nerve regeneration. Ann Anat 193(4):321–333. 10.1016/j.aanat.2011.04.008 21640570 10.1016/j.aanat.2011.04.008

[CR43] Wood MD, Kemp SWP, Liu EH, Szynkaruk M, Gordon T, Borschel GH (2014) Rat-derived processed nerve allografts support more axon regeneration in rat than human-derived processed nerve xenografts. J Biomed Mater Res A 102(4):1085–1091. 10.1002/jbm.a.34773 23630071 10.1002/jbm.a.34773

