# Correction to: Optimizing a feasible protocol for acellular nerve allografts: An experimental study

**DOI:** 10.1007/s10561-026-10216-4

**Published:** 2026-03-30

**Authors:** Marta de Juan Marín, Marta Pevida, Sara Llames, Juan Argüelles Luís, Daniel Camporro Fernández, Álvaro Meana

**Affiliations:** 1 Department of Plastic and Reconstructive Surgery, Central University Hospital of Asturias, Oviedo, Spain; 2 Department of Functional Biology, Faculty of Medicine and Health Sciences, University of Oviedo, Oviedo, Spain; 3 Clínica Alejandría, C/Sorní 12 Bajo. C.P, 46004 Valencia, Spain; 4 Unidad de Ingeniería Tisular, Centro Comunitario Sangre y Tejidos de Asturias (CCST), 33006 Oviedo, Spain; 5 Instituto de Investigación Sanitaria del Principado de Asturias (ISPA), 33011 Oviedo, Spain; 6 Centro de Investigación Biomédica en Red en Enfermedades Raras (CIBERER) ISCIII, 28029 Madrid, Spain; 7 Instituto Universitario Fernández-Vega, Fundación de Investigación Oftalmológica, Universidad de Oviedo, 33012 Oviedo, Spain; 8 Instituto de Investigación Sanitaria-Fundación Jiménez Díaz (IIS-FJD), 28015 Madrid, Spain; 9 Department of Functional Biology, Neuroscience Institute of Asturias (INEUROPA), Faculty of Medicine and Health Sciences, University of Oviedo, Oviedo, Spain

Correction to: Cell and Tissue Banking (2025) 26:49 10.1007/s10561-025-10189-w

In this article authorship order has been changed and it has been corrected as below:

1. Marta de Juan Marín.

2. Marta pevida.

3. Sara Llames.

4. Juan Argüelles Luís.

5. Daniel Camporro Fernández.

6. Álvaro Meana.

The original article has been corrected.

